# The association between the use of dry cow therapy and bacteriological cure after calving and the development of phenotypic antimicrobial resistance on Egyptian dairy farms

**DOI:** 10.1371/journal.pone.0345646

**Published:** 2026-04-01

**Authors:** Wagdy R. ElAshmawy, Noha M. Bakry, Samah M. El-Sayed, Karima M. Fahim, Samira H. Aljuaydi, Huda O. AbuBakr, Adel A. Fayed, Rehab Elhelw, Sharif S. Aly, Heba S. Farag

**Affiliations:** 1 Veterinary Medicine Teaching and Research Center, School of Veterinary Medicine, University of California Davis, Tulare, California, United States of America; 2 Department of Internal Medicine and Infectious Diseases, Faculty of Veterinary Medicine, Cairo University, Giza, Egypt; 3 Department of Food Hygiene and Control, Faculty of Veterinary Medicine, Cairo University, Giza, Egypt; 4 Department of Biochemistry and molecular biology, faculty of veterinary medicine, Cairo university, Giza, Egypt; 5 Department of Microbiology, Faculty of Veterinary Medicine, Cairo University, Giza, Egypt; 6 Department of Population Health and Reproduction, School of Veterinary Medicine, University of California, Davis, California, United States of America; Beni Suef University Faculty of Veterinary Medicine, EGYPT

## Abstract

Blanket dry cow therapy is a common practice on dairies to control mastitis, and when adopted, constitutes the majority of antimicrobial use on a dairy. The current study aimed to investigate the effect of dry cow therapy on the bacteriological cure and new infections after calving, and the association between dry cow therapy and antimicrobial resistance of *Staphylococcus aureus* isolated from milk samples of dairy cows after calving on Egyptian dairy farms. A randomized clinical trial was implemented on two Egyptian dairy farms. A total of 400 cows were randomized to one of four intramammary treatments at dry-off (Antibiotic, AB; Internal teat sealant, ITS; AB&ITS, or no treatment; Control). Composite milk samples were collected from enrolled cows at dry-off before receiving treatments and after calving. Bacterial culture and species identification were performed based on colony morphology, biochemical identification, and PCR using 16S rRNA. The Minimum Inhibitory Concentration (MIC) for each of the study *Staphylococcus aureus* (*S. aureus*) isolates was estimated using a commercial plate (CMV1AMAF Sensititre plate). Logistic regression models were used to estimate the effect of the different dry cow treatments on bacteriological cure and new infections. Parametric survival interval regression models were used to model the association between dry cow treatment and antimicrobial resistance after calving. Cows with a history of mastitis at the dry-off lactation had significantly higher odds (Odds Ratio, OR = 2.2, SE = 0.005, P < 0.01) of bacteriological cure compared to cows with no history of mastitis at the dry-off lactation. The highest percentage of resistance at dry-off was observed against penicillin (64%), sulfadimethoxine (54%), and erythromycin (30%) during the Spring/Summer season, while 37% was observed against penicillin and 30% for sulfadimethoxine during the Fall/Winter season. There was a significant increase in the MIC for ampicillin and ceftiofur in *S. aureus* isolated from cows that received AB and AB&ITS at dry-off in both study seasons. The current study revealed that more than 30% of subclinical mastitis cows were infected with *S. aureus* at dry-off and after calving, which requires future studies to evaluate the control measures of contagious mastitis implemented on Egyptian dairies before implementing the selective dry cow therapy algorithm.

## 1. Introduction

Antimicrobial resistance is a worldwide growing threat to the health and well-being of humans, animals, plants, and the environment [1]. The worldwide estimated number of deaths associated with antimicrobial resistance was 4.95 million in 2019 [2]. To keep the effectiveness of antimicrobials, regulations have been implemented to reduce the unnecessary use of antimicrobials and improve the antimicrobial stewardship programs to improve public awareness and increase knowledge on the judicious use of antimicrobials [3].

The major use of antimicrobials on dairies was to treat and control mastitis [4]. According to the 2013 United States Department of Agriculture (USDA) report, 99.7% of dairy operations reported at least one case of mastitis, and more than 96.9% included antimicrobials in their treatment protocols. Dry cow therapy has been used since the late 1960s as one of the five-point plan to control mastitis in dairy cows, which has now been advanced to the 10-point plan by the National Mastitis Council [5]. Currently, blanket dry cow therapy is the common practice on dairy farms through injecting each quarter at dry-off with an intramammary antibiotic tube [6]. More than 80% of the dairy farms in the USA are using blanket dry cow therapy [7], but with the growing concern of the development of antimicrobial resistance worldwide, strategies have been implemented to reduce the unnecessary use of antimicrobials on dairy farms. Dry cow therapy is a major area for antimicrobial use on dairy farms, and recent research found that not every dairy cow requires dry cow treatment. In the last two decades, researchers have been advocating for the selective dry cow therapy (SDCT) through developing algorithms to identify cows under high risk of developing mastitis during the dry-off or in the subsequent lactation to target with the dry cow antimicrobial tubes [8,9].

Researchers investigated the development of antimicrobial resistance of mastitis pathogens isolated from mastitis milk samples [10,11], but few studies have researched the effect of using dry cow therapy on the development of antimicrobial resistance of milk mastitis pathogens after calving [12,13]. None of these studies was done in Egypt. The current study aimed to 1) study the effect of using dry cow therapy on the bacteriological cure and the development of new bacterial infections after calving, 2) identify the bacteriological profile of mastitis pathogens isolated at dry-off, after calving and the first mastitis event within the first 60 days in milk (DIM) from dairy cows in Egypt, 3) study the association between the use of dry cow therapy and the development of phenotypic antimicrobial resistance of the *Staphylococcus aureus* isolates in dairy cows after calving. The outcomes of the current study will highlight the AMR development due to the use of dry cow therapy and its impact on bacteriological cure and the development of new bacterial infections in the subsequent lactation.

## 2. Materials and methods

### 2.1. Study design and herd enrollment

A randomized field trial was conducted on two Egyptian dairy farms between February 2023 and November 2024. A total of 400 cows were enrolled in two seasonal cohorts (Fall/Winter and Spring/Summer) and followed up until 120 days in milk (DIM). The dairies were located in El Fayoum and Dakahliya governorates in Egypt. Enrolled cows were randomized to receive one of the four treatments at dry-off: intramammary antimicrobial infusion (AB), internal teat sealant (ITS), both (AB&ITS), or no treatment (control), with 100 cows in each treatment group. The study was approved by the Cairo University IACUC committee (protocol number VetCU10102019086) and the University of California, Davis’s IACUC committee (protocol number 21383) on December 17th, 2021.

### 2.2 Cow enrollment and sampling procedures

At each dairy visit, all dairy cows that had not received any antibiotic treatments within the last 30 days of enrollment on the dry-off list were visually examined, and only healthy cows with no signs of lameness, had a good body condition score (BCS > 2.5), and did not have clinical mastitis at dry-off were eligible for enrollment. After their last milking, before dry-off, all eligible cows were randomly assigned to one of the four treatments (AB, ITS, AB&ITS, or Control); random assignment was generated in Excel (Microsoft Corp., Redmond, WA). Enrolled cows were examined for their udder hygiene (UHS) using a four-point scoring system (1 is clean and 4 is dirty) and similarly the teat end score (TES), ranging from one being normal and four if a quarter has a rough and cracked teat end [14]. The California Mastitis Test (CMT) was performed at enrollment, and the scores were recorded on a scale of 0, trace, 1,2, or 3 with increasing gel formation as the scores increased [14].

Aseptic milk samples were collected from cows at enrollment and before administration of the dry-off treatment. After recording the UHS, TES, and CMT, the teat end was cleaned with 70% alcohol wipes, the first 2–3 strips discarded, and a composite milk sample was collected from each cow. In the current study, we used composite milk samples as they are cost-effective in testing large numbers of samples [15] and have shown similar specificity to the quarter milk samples but lower sensitivity [16]. Milk samples were collected in duplicates. A second sample was collected from enrolled cows at 3–7 days in milk, following the same procedure. Cows that had mastitis within the first 60 days after calving had a milk sample collected from the affected quarter before being treated.

### 2.3. Bacterial culture and identification

Milk samples were cultured for bacterial isolation and identification at the Infectious Disease laboratory, Faculty of Veterinary Medicine, Cairo University, Giza, Egypt, following the NMC guidelines [17]. Colony types were identified by colony morphology, hemolysis, Gram staining, and biochemical tests. *Staphylococcus aureus* (*S. aureus*) was identified using the coagulase test and confirmed using polymerase chain reaction (PCR) using 16S rRNA. In addition to direct plating through surface spreading of milk on Hayflick medium for *Mycoplasma* species, milk samples were tested using enrichment broth incubated at 37°C for 24 hours, followed by plating on Mycoplasma agar plates [18]. The plates were incubated in a 5–10% CO2 incubator and examined daily for 7 days before being reported as negative.

The bacterial isolates were subjected to molecular identification using PCR targeting specific genes for non-aureus staphylococci (NAS), *Streptococcus* spp., *E. coli*, and *Klebsiella* species. *Staphylococcus* strains were identified using 16S rRNA and nuc gene primers [19], while a multiplex PCR approach was used for *Streptococcus* species as described in Raemy et al., 2013 [18]. The 16S rRNA gene was targeted for *E. coli* identification, and *Klebsiella* isolates were confirmed using the gyrA gene [20]. The PCR primers used for the identification of different bacterial isolates are listed in Table S1 Table. The bacterial DNA was extracted from all obtained isolates using a QIAamp DNA Mini Kit (Qiagen®, Germany) following the manufacturer’s instructions. The *S. aureus* (ATCC® 6538P) and *E. coli* (ATCC® 8739) were used as reference strains.

### 2.4. Antimicrobial susceptibility testing

The antimicrobial susceptibility was performed only on the *S. aureus* isolates (dry-off, fresh, mastitis samples). The minimum inhibitory concentration (MIC) was determined using the microbroth dilution assay using commercial mastitis-specific plates (CMV1AMAF ®; Sensititre, Thermofisher) following the manufacturer’s instructions. Reading of the plates was done using the Sensititre TM Vizion TM Digital MIC Viewing System. The MIC cut-offs were used following previous literature [10,21].

### 2.5. Statistical analysis

Descriptive statistics for the results of bacterial cultures for different treatment groups (AB, ITS, AB&ITS, control) at dry-off and fresh sampling points in different seasons were summarized. The frequency of *S. aureus* isolates at different MICs isolated from dry-off samples, fresh, and clinical mastitis samples during both seasons was similarly summarized. All statistical analysis was performed using Stata statistical software release 18 (Stata Corp LLC, TX, USA).

#### 2.5.1 Modeling of the Bacteriological Cure and New Bacterial Infections.

A logistic regression model was used to model the bacteriological cure (Yes/No) at the cow level and accounting for clustering by farm using the variance–covariance matrix of the estimators (vce). Intramammary infection (IMI) cure was determined by comparing the results of bacterial culture of milk samples collected at dry-off (S1) and at freshening (S2). Intra-mammary infection cure (Yes) was determined if a cow had a positive culture result at dry-off, followed by a negative result after freshening for the same organism. Conversely, no IMI cure (No) was determined if a cow had a culture-positive milk sample for the same pathogen type at both dry-off and freshening. Cows with contaminated milk samples at dry-off or freshening were excluded from this analysis. The treatment variable was forced into all the models as the main goal was to evaluate the effect of different treatments (AB, ITS, AB&ITS, Control) on the bacteriological cure. Different confounders, including parity, season, udder hygiene score, teat end score, and CMT score at enrollment, were assessed. A manual backward approach was used for model building and known confounders and effect modifiers were assessed using the change in estimates and significance testing [22]. Covariates were retained in the final model if the P-value was < 0.05. The best fit model was selected based on the lowest Akaike information criterion (AIC) for the better model goodness of fit [23].

Similarly, a logistic regression model was used to model new bacterial infections (presence of new infection, Yes/No) at the cow level and accounting for clustering by farm using the variance–covariance matrix of the estimators (vce). New bacterial infections were determined by comparing bacterial culture results of milk samples after freshening to bacterial culture results of milk samples collected at the time of enrollment (dry-off). New bacterial infections (Yes) were interpreted as the presence of new or different bacterial growth on the dry-off milk samples in comparison to fresh samples. No new bacterial infections (No) status was determined if a cow had the same milk culture results at dry-off and after freshening. Cows with contaminated milk samples at dry-off or at freshening were excluded from the analysis. Confounders were accounted for, and the final model was developed by following the same procedures described previously in the bacteriological cure section.

#### 2.5.2 Modelling the effect of different treatments at dry-off on the MIC Values after calving.

The modeling was limited to cows with *S. aureus* isolated at both dry-off and after calving. The main goal was to evaluate the effect of different dry-off treatments (AB, ITS, AB&ITS, Control) on the MIC values of different antimicrobials of the CMV1AMAF plate (ampicillin, ceftiofur, erythromycin, oxacillin, penicillin, penicillin/novobiocin, pirlimycin, tetracycline, cephalothin, Sulfadimethoxine). Parametric survival interval regression models with accelerated failure time parametrization were used to model the effect of different treatments on the MIC values for the *S. aureus* isolates after calving, as previously described in Okello et al., 2022 [12]. Briefly, the MIC values were treated as censored data, with the upper value, which represented the highest value of the MIC (maximum MIC value) on the plate, censored to the right (∞), and the lower value, which represented the lowest MIC, was assigned to half of the lowest MIC value on the plate. Confounders were adjusted for in the model, including parity, season, mastitis history during the dry period, TES, and CMT score. To account for the potential non-normality of MIC distributions, different distributions, including exponential, Weibull, Gompertz, lognormal, loglogistic, or generalized gamma distributions, were explored. The best model fit was based on the lowest AIC [23]. Model estimates were reported after exponentiation allowing their interpretation as ratios of the MIC in *S. aureus* isolates from milk samples of cows treated at dry off with one of the study treatments compared to those from controls, after adjusting for any other variables in the final model.

## 3. Results

### 3.1. Different bacterial isolates at dry-off, after calving, and clinical mastitis

Results of the bacterial culture of composite milk samples collected at dry-off, after freshening during the Fall/Winter and Spring/Summer seasons, and at the first event of clinical mastitis during the first 60 DIM of the subsequent lactation are summarized in Table 1, S2, and S3 and S4 Tables. A total of 400 samples were cultured at dry-off; of those, 70% had single colony culture (pure culture), 23% had no bacterial growth, 6% had mixed infections (had growth of 2 different colonies), and 1% were contaminated (had growth of more than 3 different bacterial species). The bacterial isolates of the pure culture were 58% NAS, 37.5% *S. aureus*, 2% *E. coli*, and 1.5% and 1% for each of *Klebsiella* spp. and *Streptococcus agalactiae*. After calving, a total of 380 samples were cultured as 20 cows were culled (15 cows), aborted (4 cows), or died (1 cow) during the dry period. There were 23% of the samples yielded no bacterial growth, 68% had pure culture, 8% had mixed infections, and 1% contaminated. The percentage of bacteria isolated from the pure culture was 60% NAS, 37% *S. aureus*, 1.5%, 1%, and 1% for *Streptococcus agalactiae*, *Klebsiella* spp., and *Streptococcus dysgalactiae*.

**Table 1:** The percentage of different bacterial isolates isolated from milk samples collected at dry-off and after calving during Fall/Winter and Spring/Summer seasons.

Bacterial Isolates	Dry-off milk samples			Fresh milk samples		
	Fall/Winter (%)	Spring/Summer (%)	Total (%)	Fall/Winter (%)	Spring/Summer (%)	Total (%)
Non-aureus staphylococci	56	61	58	60	59	60
*Staphylococcus aureus*	40	36	37.5	37	37	37
*E. coli*	2	2	2	0	1	0.5
*Klebsiella species*	2	1	1.5	1	1	1
*Streptococcus agalactiae*	1	1	1	1	2	1.5
*Streptococcus dysgalactiae*	0	0	0	1	1	1

A total of 62 cows had clinical mastitis during the first 60 DIM, and the most frequent bacterial species isolated were *S. aureus* (37.1%), followed by NAS and *E. coli* 29% each, and 4.9% were *Streptococcus agalactiae*. The data of the different isolates from different seasons and for different treatment groups are shown in S3, S4, and S5 Tables. *Mycoplasma* spp. was not isolated from any of the dry-off, after calving, or clinical mastitis milk samples.

### 3.2 Bacteriological cure and new bacterial infections

Results of modelling bacteriological cure are presented in Table 2. The history of mastitis at dry-off lactation had a modifying effect on dry cow treatment. There was no significant association between dry cow treatments (AB, ITS, AB&ITS) and the bacteriological cure for cows with no history of mastitis in the dry-off lactation. In contrast, cows with a history of mastitis at dry-off lactation had higher odds (2.2 times) of bacteriological cure in comparison to cows with no mastitis at dry-off lactation. Cows with a history of mastitis at dry-off lactation that received ITS had lower odds of bacteriological cure (0.87, P < 0.01) in comparison to cows that did not receive any treatment. In contrast, cows with a history of mastitis at dry-off lactation that received AB or AB&ITS had higher odds of bacteriological cure (1.95 and 1.22, respectively), but the association was not significant (P > 0.05).

**Table 2:** Logistic regression model for predicting bacteriological cure after freshening for cows with positive bacteriological cultures at dry-off among different treatment groups (AB, ITS, ITS&AB, control).

Variable	Levels	Coefficient	SE*	P-value	95% CI**
Treatment	Control (No treatment)	Referent			
	Intramammary antibiotics	−0.35	0.313	0.25	(−0.96, 0.26)
	Internal teat sealants	0.19	0.174	0.27	(−0.14, 0.53)
	Both intramammary antibiotics and teat sealants	0.07	0.369	0.83	(−0.64, 0.80)
Mastitis in the dry-off lactation	No	Referent			
	Yes	0.79	0.002	< 0.01	(0.78, 0.79)
Interaction between treatment and mastitis at dry-off lactation	Received no treatment and had no mastitis at dry-off	Referent			
	Received Intramammary antibiotics and had mastitis at dry-off	0.23	0.146	0.108	(- 0.05, 0.52)
	Received Internal teat sealants and had mastitis at dry-off	−1.11	0.174	< 0.01	(−1.44, −0.76)
	Received both intramammary antibiotics and internal teat sealants, and had mastitis at dry-off	−0.66	0.769	0.39	(−2.17, 0.84)
Intercept		0.30	0.002	< 0.01	(0.30, 0.31)

*SE: Standard Error

**CI: Confidence interval

Results of modelling new infections are presented in Table 3. There was no significant association between dry cow treatments (AB, ITS, AB&ITS) and new bacterial infections. Third-lactation cows had significantly higher odds of developing new infections in comparison to second-lactation cows (1.85, P < 0.01). Cows with a sum of the CMT score of 8 or greater for the four quarters had significantly higher odds of developing new infections in comparison to cows with a sum of CMT score lower than 8 (4.22, P < 0.01).

**Table 3:** Logistic regression model for predicting new bacteriological infections after freshening for cows with positive bacteriological cultures at dry-off among different treatment groups (AB, TS, TS&AB, control).

Variable	Levels	Coefficient	SE*	P-value	95% CI**
Treatment	Control (No treatment)	Referent			
	Intramammary antibiotics	0.90	0.618	0.89	(0.75, −0.02)
	Internal teat sealants	0.81	0.274	0.54	(−1.18, −1.16)
	Both intramammary antibiotics and teat sealants	0.80	0.231	0.44	(−0.59, −0.58)
Parity (lactation number)	2	Referent			
	3	0.62	0.017	< 0.01	(0.59, 0.66)
	>3	0.65	0.167	0.09	(0.39, 1.07)
Sum of CMT scores	< 8	Referent			
	≥ 8	1.44	0.054	< 0.01	(1.34, 1.55
Intercept		1.91	0.499	0.01	(1.14, 3.19)

*SE: Standard Error

**CI: Confidence interval

### 3.3. Phenotypic antimicrobial resistance of *Staphylococcus aureus*

The MIC values for the *S. aureus* isolates from milk samples collected at dry-off and after calving for Fall/Winter and Spring/Summer seasons are presented in Tables 4-7, respectively. The percentage of AMR-resistant *S. aureus* isolates was higher for the dry-off and fresh samples during the Spring/Summer season in comparison to the Fall/Winter season. The highest percentage of resistance during dry-off isolates was reported for penicillin (64%), sulfadimethoxine (54%), and erythromycin (30%) during the Spring/Summer season, while 37% was reported for penicillin and 30% for sulfadimethoxine during the Fall/Winter season. The percentage of isolates with AMR increased in the *S. aureus* isolates isolated from milk samples after freshening in comparison to the dry-off isolates. For the Spring/Summer season, the highest percentage of resistance after freshening were 72% and 66% against penicillin and ampicillin, respectively, in comparison to 64% and 28% at dry-off. A similar trend was also observed in the Fall/Winter season, with 55% and 42% resistance against penicillin and ampicillin, respectively, in comparison to 37% and 21% at dry-off. The MIC values for the *S. aureus* isolates from different treatment groups at dry-off and fresh isolates are summarized in supplemental S6-S13 Tables. The majority of the isolates had higher resistance in the fresh isolates in comparison to the dry-off isolates before receiving the dry-off treatment.

**Table 4:** The percentage of isolates at each minimum inhibitory concentration (MIC) for different antimicrobials for the *Staphylococcus aureus* isolates from the dry-off milk samples collected during the Fall/Winter season. Bold estimates represent the respective antimicrobial drug cut-off for resistance.

Antimicrobial/MIC* Values (µg/mL)	0.12	0.25	0.5	1	2	4	8	16	32	64	128	256	MIC 50	MIC 90
Ampicillin	56	23	**5**	7	4	0	5						0.12	1.00
Penicillin	63	**16**	5	9	0	0	7						0.12	1.00
Erythromycin		42	42	2	0	**14**							0.50	4.00
Ceftiofur			46	42	5	**7**							1.00	2.00
Pirlamycin			75	9	5	**11**							0.50	4.00
Pencillin/Novobiocin				96	4	**0**	0						1.00	1.00
Tetracycline				88	4	2	**7**						1.00	2.00
Cephalothin					95	4	2	0					2.00	2.00
Oxacillin					89	**11**							2.00	4.00
Sulfadimethoxine									58	11	2	**30**	32.00	≥ 256

**Table 5:** The percentage of isolates at each minimum inhibitory concentration (MIC) for different antimicrobials for the *Staphylococcus aureus* isolates from the fresh milk samples collected during the Fall/Winter season. Bold estimates represent the respective antimicrobial drug cut-off for resistance.

Antimicrobial/MIC* values (µg/mL)	0.12	0.25	0.5	1	2	4	8	16	32	64	128	256	MIC 50	MIC 90
Ampicillin	50	9	**6**	15	6	2	13						0.12	8.00
Penicillin	44	**11**	9	7	2	7	19						0.25	8.00
Erythromycin		52	30	2	4	**13**							0.25	4.00
Ceftiofur			37	33	7	**22**							1.00	4.00
Pirlamycin			74	6	7	**13**							0.50	4.00
Pencillin/Novobiocin				91	6	**0**	4						1.00	1.00
Tetracycline				69	4	6	**22**						1.00	8.00
Cephalothin					74	4	2	20					2.00	16.00
Oxacillin					76	**24**							2.00	4.00
Sulfadimethoxine									52	9	2	**37**	32.00	≥ 256

**Table 6:** The percentage of isolates at each minimum inhibitory concentration (MIC) for different antimicrobials for the *Staphylococcus aureus* isolates from the dry-off milk samples collected during the Spring/Summer season. Bold estimates represent the respective antimicrobial drug cut-off for resistance.

Antimicrobial/MIC values (µg/mL)	0.12	0.25	0.5	1	2	4	8	16	32	64	128	256	MC 50	MC 90
Ampicillin	25	48	**3**	7	8	5	5						0.25	2.00
Penicillin	34	**26**	11	3	5	3	16						0.25	8.00
Erythromycin		44	20	5	2	**30**							0.50	4.00
Ceftiofur			30	44	7	**20**							1.00	4.00
Pirlamycin			64	8	2	**26**							0.50	4.00
Pencillin/Novobiocin				89	10	**0**	2						1.00	2.00
Tetracycline				74	5	2	**20**						1.00	8.00
Cephalothin					82	7	1	6					2.00	4.00
Oxacillin					77	**23**							2.00	4.00
Sulfadimethoxine									31	13	2	**54**	≥ 256	≥ 256

**Table 7:** The percentage of isolates at each minimum inhibitory concentration (MIC) for different antimicrobials for the *Staphylococcus aureus* isolates from the fresh milk samples collected during the Spring/Summer season. Bold estimates represent the respective antimicrobial drug cut-off for resistance.

Antimicrobial/MIC* values (µg/mL)	0.12	0.25	0.5	1	2	4	8	16	32	64	128	256	MIC 50	MIC 90
Ampicillin	23	11	**21**	15	6	13	11						0.50	8.00
Penicillin	28	**11**	6	4	9	21	21						1.00	8.00
Erythromycin		45	36	6	2	**11**							0.50	4.00
Ceftiofur			23	23	17	**36**							2.00	4.00
Pirlamycin			87	2	4	**6**							0.50	4.00
Pencillin/Novobiocin				94	2	**0**	2						1.00	1.00
Tetracycline				55	2	0	**20**						1.00	1.00
cephalothin					68	6	0	26					2.00	8.00
Oxacillin					64	**36**							2.00	16.00
Sulfadimethoxine									38	11	4	**47**	128.00	≥ 256

### 3.3. Effect of different dry cow treatments on the MIC values after calving

Results of the parametric survival models for the effect of different dry cow treatments on the MIC values for different antimicrobials on the commercial mastitis Sensititre plate CMV1AMAF® against *S. aureus* isolated from milk samples at dry-off and after calving are presented in Table 8. The results showed a positive trend in the MIC values for all the antimicrobials for the isolates of the Spring/Summer season in comparison to the Fall/Winter season. There is a significant positive trend in the MIC values for *S. aureus* reported for cows that received AB and AB&ITS at dry-off against ceftiofur for both seasons. There was a significant positive trend for cows that received both AB and AB&ITS at dry-off for the Spring/Summer season against Ampicillin, cephalothin, and sulfadimethoxine. There was a significant negative trend for the MIC values for cows infected with *S. aureus* at the Fall/Winter season for cows that received ITS for the MIC values against erythromycin, tetracycline, oxacillin, and sulfadimethoxine, and there was a significant positive trend for the same isolates against cephalothin, penicillin/Novobiocin, and pirlimycin during the same season. During the Spring/Summer season, *S. aureus* isolates from cows received ITS at dry-off showed a significant positive trend for ampicillin, erythromycin, ceftiofur, pirlimycin, Penicillin/Novobiocin, tetracycline, cephalothin, and sulfadimethoxine.

**Table 8:** Parametric survival interval regression models for predicting the antimicrobial resistance against *Staphylococcus aureus* isolated from cows at dry-off and after calving.

Antimicrobial Drug		Season 1 (Fall/Winter)			Season 2 (Spring/Summer)		
		Intramammary antibiotics	Internal teat sealants	Both	Intramammary antibiotics	Internal teat sealants	Both
Ampicillin^a^	MIC Value	1.29*	2.05	1.43	2.28*	3.62*	2.52*
	95% CI	(1.26, 1.33)	(0.002, 4.11)	(0, 2.96)	(1.35, 3.22)	(1.37, 5.86)	(0.79, 4.25)
Penicillin^a^	Coefficient	01.25*	3.27	1.68	2.65*	6.96	3.57
	95% CI	(1.05, 1.44)	0, 10.58)	(0, 3.94)	(2.58, 2.72)	(0, 21.63)	(00, 7.94)
Erythromycin	Coefficient	0.75*	0.96*	0.89*	0.96*	1.23*	1.15*
	95% CI	(0.23, 1.28)	(0.89, 1.04)	(0.85, 0.94)	(0.27, 1.66)	(1.18, 1.29)	(1.05, 1.24)
Ceftiofur^a^	Coefficient	1.09*	1.40	1.53*	1.59*	2.03*	2.23*
	95% CI	(1.08, 1.10)	(0, 2.81)	(0.77, 2.29)	(1.31, 1.87)	(0.34, 3.71)	(1.53, 2.92)
Pirlimycin	Coefficient	0.89*	1.02*	1.09*	0.92*	1.06*	1.13*
	95% CI	(0.59, 1.11)	(0.79, 1.26)	(1.04, 1.15)	(0.51, 1.32)	(0.72, 1.41)	(0.97, 1.30)
Penicillin/ Novobiocin	Coefficient	0.96*	1.02*	1.02*	0.99*	1.05*	1.05*
	95% CI	(0.90, 1.02)	(0.99, 1.05)	(0.87, 1.19)	(0.81, 1.17)	(0.95, 1.16)	(0.77, 1.35)
Tetracycline	Coefficient	0.71*	0.77*	0.67*	0.96*	1.05*	0.92*
	95% CI	(0.13, 1.28)	(0.74, 0.80)	(0.27, 1.07)	(0.45, 1.46)	(0.79, 1.31)	(0.63, 1.20)
Cephalothin	Coefficient	0.93*	1.13*	1.03*	1.03*	1.25*	1.15*
	95% CI	(0.74, 1.12)	(1.10, 1.15)	(0.81, 1.26)	(0.85, 1.22)	(1.19, 1.31)	(0.93, 1.37)
Oxacillin	Coefficient	0.97*	0.991*	0.99*	0.99*	1.01	1.01*
	95% CI	(0.93, 1.01)	(0.991, 0.992)	(0.98, 1.01)	(0.93, 1.03)	(0.99, 1.02)	(0.99, 1.03)
Sulfadimethoxine	Coefficient	0.52*	0.83*	0.70*	1.02*	1.62*	1.37*
	95% CI	(0.37, 0.6	(0.44, 1.20)	(0.19, 1.21)	(0.75, 1.30)	(0.91, 2.33)	(0.40, 2.35)

* Significant, P value < 0.05

^a^These models were adjusted for parity

## 4. Discussion

The current study reported 70% of the milk samples had single colony culture (pure culture), 23% had no bacterial growth, 6% had mixed infections, and 1% were contaminated. The common bacterial isolates were NAS (58%) and *S. aureus* (37.5%). The common bacterial isolates for the clinical mastitis samples collected during the first 60 days after calving revealed that 37.1% were *S. aureus*, 29% were *E. coli*, and 4.9% were *Streptococcus agalactiae*. These results indicate that the percentage of contagious mastitis pathogens (*S. aureus* and *Streptococcus agalactiae*) was high in the study herds, which may require more aggressive strategies to control the contagious mastitis problem before considering the application of SDCT [28]. The study results are comparable to the percentage of *S. aureus* (35–45.7%) isolated from other studies done in Egypt, but their study was done on cows raised individually [29] and 36.7% in fresh cow milk samples randomly collected from dairy farms [30]. *Mycoplasma* spp. was not detected in the dry-off, after caving, or mastitis milk samples within the first 60 days after calving. Our results are in contrast with Abd El Tawab et al. (2019), who reported a prevalence that ranged from 3.6% to 28.6% in Giza and Al-Buhayrah Governorates in Egypt, and Saad and Abdel Hameed (2012), who isolated *Mycoplasma bovirhinis* from 6.7% of cow’s milk samples in Qena, Egypt [31,32]. Absence of *Mycoplasma* spp. as a contagious mastitis pathogen in our herds could have been related to our study’s different Governorates, or that the majority of our samples (dry-off samples and after calving) were collected from non-mastitic cows in comparison to clinical mastitis samples in Abd El Tawab et al. (2019).

The current study investigated the association between the different dry cow therapy treatments and the bacteriological cure and development of new bacterial infections after calving on two Egyptian dairies at two different seasons (Fall/Winter and Spring/Summer). The current trial employed composite samples, which have been shown to have similar specificity but lower sensitivity, in comparison to quarter milk samples [16]. Despite the differences in sensitivity, analyses of the trial outcomes identified significant differences between treatment groups. Despite composite samples being an aggregate sample, similar to pooled samples, they may be more cost-effective than testing quarter samples [15]. The current study reported that only cows with a history of mastitis at dry-off lactation had higher odds of bacteriological cure compared to cows with no mastitis at dry-off lactation. This finding requires further investigation with future studies focusing on cows with a history of mastitis, as our study had a lower number of cows with a history of mastitis at dry-off lactation. These results indicate that cows with a history of mastitis in the dry-off lactation may be at higher risk of having bacterial infections after calving, hence showed benefit from the application of long-acting antimicrobial dry cow tubes at dry-off, as reported in previous studies [24–26]. The study reported that ITS alone was not effective in promoting the bacteriological cure, as cows with a history of mastitis at dry-off lactation that received ITS had lower odds of bacteriological cure (0.87, P < 0.01) in comparison to cows with no history of clinical mastitis that did not receive any treatments at dry-off. That outcome agreed with the decision made by previous researchers designing their selective dry cow therapy algorithm not to apply the selective dry cow therapy (SDCT) to cows with a previous mastitis history [22–25]. The current study reported no significant association between different dry cow treatments (AB, ITS, AB&ITS) and the bacteriological cure for cows with no history of mastitis in the dry-off lactation, and these results indicate that SDCT is a good option for these cows. Previous studies reported no significant difference in the bacteriological cure after calving for cows with no history of mastitis during dry lactation [24,27]. The study did not find any significant association between different dry cow treatments (AB, ITS, AB&ITS) and the new bacterial infections, but third-lactation cows had a significantly higher odds of having new infections after calving in comparison to second-lactation cows (1.85, P < 0.01). In addition, cows with higher CMT scores at dry-off had a significantly higher odds of having new infections after calving in comparison to cows with lower CMT scores. These results indicate that older cows and cows with higher CMT scores are at higher risk of developing new infections, and these cows may require antimicrobial treatments at dry-off as recommended by previous studies [26,27].

The current study investigated the association between the different dry cow therapy treatments and the development of AMR for the *S. aureus* isolated after calving from two Egyptian dairies at two different seasons (Fall/Winter and Spring/Summer). The percentage of AMR-resistant *S. aureus* isolates was higher for the dry-off and fresh samples during the Spring/Summer season in comparison to the Fall/Winter season, with the highest percentage of resistance during dry-off isolates was reported for penicillin (64%), sulfadimethoxine (54%), and erythromycin (30%). The percentage of isolates with AMR increased in the *S. aureus* isolates isolated from the fresh milk samples in comparison to the dry-off isolates, with the highest percentage reported 72% and 66% against penicillin and ampicillin, respectively, in comparison to 64% and 28% for dry-off isolates. These percentages are higher than what was reported in a different Egyptian study that reported a resistance of 54.5%, 45.5%, and 20.5% against penicillin, ampicillin, and erythromycin [28]. The majority of the isolates had higher resistance in the fresh isolates in comparison to the dry-off isolates, which may indicate that the dry cow treatment impacted the resistance profile for the *S. aureus* isolates. A previous study reported an increase in the trend of resistance in the NAS isolates from the fresh milk samples in comparison to the dry-off samples [12].

### Limitations

The current study was performed on only two Egyptian dairies, which may not represent all the different management practices in Egyptian milk sheds. None of the study dairies were testing for the somatic cell counts, which may limit the ability to diagnose the sub-clinical mastitis after calving. The CMT was done only at enrollment (dry-off), which limits evaluating the effect of different dry cow treatments on the cure of subclinical mastitis cases. Intramammary infection status at dry off was determined at the cow level due to the collection of composite milk samples at dry off and post-calving. Hence, IMI frequency and model results should be interpreted with caution due to the potential for misclassification error.

## 5. Conclusions

The current study revealed that antimicrobial dry-off tubes improved the bacteriological cures of cows with a history of mastitis at dry-off lactation. *Staphylococcus aureus* was the common contagious mastitis isolated from more than 30% of the non-clinical mastitis cows at dry-off and after calving, and this will require future studies to evaluate the control measures of contagious mastitis implemented on Egyptian dairies before implementing the SDCT. There was an increase in the antimicrobial resistance of *S. aureus* isolates from the fresh samples in comparison to dry-off samples, especially for Spring/Summer season samples.

**Informed consent statement:** No human patients were involved in the study.

## Supporting information

S1 Table Primer names, target genes, oligonucleotide sequences, and the product size used in different PCR assays. (DOCX)

S2 Table The percentage of bacterial no growth, pure colonies, mixed infections, and contaminated milk samples collected at dry off and after freshening. (DOCX)

S3 Table The percentage of different bacterial isolates isolated from clinical mastitis milk samples during the first 60 days in milk during the Fall/Winter and Spring/Summer seasons. (DOCX)

S4 Table The percentage of different bacterial isolates isolated from clinical mastitis milk samples during the first 60 days for different treatment groups. (DOCX)

S5 Table The percentage of mixed infections, by bacterial species, as isolated from milk samples at collected at dry off and after calving from two Egyptian dairies over the Fall/Winter and Spring/Summer seasons. (DOCX)

S6 Table The percentage of isolates at each minimum inhibitory concentration (MIC) for different antimicrobials for the *Staphylococcus aureus* isolates from the dry off milk samples for the group that received intramammary antibiotics. (DOCX)

S7 Table The percentage of isolates at each minimum inhibitory concentration (MIC) for different antimicrobials for the *Staphylococcus aureus* isolates from the fresh milk samples for the group that received intramammary antibiotics. (DOCX)

S8 Table The percentage of isolates at each minimum inhibitory concentration (MIC) for different antimicrobials for the *Staphylococcus aureus* isolates from the dry off milk samples for the group that received intramammary antibiotics and internal teat sealants at dry off. (DOCX)

S9 Table The percentage of isolates at each minimum inhibitory concentration (MIC) for different antimicrobials for the *Staphylococcus aureus* isolates from the fresh milk samples for the group that received intramammary antibiotics and internal teat sealants at dry off. (DOCX)

S10 Table The percentage of isolates at each minimum inhibitory concentration (MIC) for different antimicrobials for the *Staphylococcus aureus* isolates from the dry off milk samples for the group that received internal teat sealants at dry off. (DOCX)

S11 Table The percentage of isolates at each minimum inhibitory concentration (MIC) for different antimicrobials for the *Staphylococcus aureus* isolates from the fresh milk samples for the group that received internal teat sealants at dry off. (DOCX)

S12 Table The percentage of isolates at each minimum inhibitory concentration (MIC) for different antimicrobials for the *Staphylococcus aureus* isolates from the dry off milk samples collected during the Fall/Winter season for the control group. (DOCX)

S13 Table The percentage of isolates at each minimum inhibitory concentration (MIC) for different antimicrobials for the *Staphylococcus aureus* isolates from the fresh milk samples collected during the Fall/Winter season for the control group. (DOCX)

S1 File Bacterial culture data. (XLS)
