# Supplementary material for: The association between the use of dry cow therapy and bacteriological cure after calving and the development of phenotypic antimicrobial resistance on Egyptian dairy farms
Source: PLoS One. 2026 Apr 1;21(4):e0345646. doi: 10.1371/journal.pone.0345646 (PMC13043046; doi:10.1371/journal.pone.0345646)
Supplement: S1 Table — (DOCX) [file pone.0345646.s001.docx]

**Table S1. Primer names, target genes, oligonucleotide sequences, and the product size used in different PCR assays**

| **Primer name (target gene)** | **Oligonucleotide sequences (5′–3′)** | **Product size (bp)** | **References** |
| --- | --- | --- | --- |
| *Staphylococcus spp.*  (16SrRNA) | F: GTA GGT GGC AAG CGT TAT CC  R: CGC ACA TCA GCG TCA G | 228 | [1] |
| *S.aureus*  (au-F3  au-nucR) | F: ACAGAGTTAGAGCATCAATCAC  R: GACCACCGCCATTATTACG | 433 | [2] |
| *Streptococcus uberis (pauA)* | F: TGA TTC CGA CTA CTA CGC TAG AT  R: ATA CTT TGA GTT TCA CCG AGT TC | 723 | [3] |
| *Streptococcus dysgalactiae (16S rRNA)* | F: GTG CAA CTG CAT CAC TAT GAG  R: CGT CAC ATG GTG GAT TTT C | 279 |  |
| *Streptococcus agalactiae (sklA3)* | F:ATT GAT AAC GAC GGT GTT ACT GT  R: CAT AGT AGC GTT CTG TAA TGA TGT C | 487 |  |
| *E. coli*  (*16S rRNA*) | F: CCCCCTGGACGAAGACTGAC  R: ACCGCTGGCAACAAAGGATA | 401 | [4] |
| *Klebsiella spp. (gyrA*) | F-CGCGTACTATACGCCATGAACGTA  R-ACCGTTGATCACTTCGGTCAG G | 441 | [5] |

[1] Monday, S.R. and Bohach, G.A. (1999). Use of multiplex PCR to detect classical and newly described pyrogenic toxin genes in staphylococcal isolates. J. Clin. Microbiol., 37(10):3411-3414.

[2] Schissler, J.R. (2009). Species Identification by Polymerase Chain Reaction of Staphylococcal Isolates from the Skin and Ears of Dogs and Evaluation of Clinical Laboratory Standards Institute Interpretive Criteria for Canine Methcillin-resistant Staphylococcus pseudointermedius. M.V.Sc. Thesis, Ohio State University. 151p.

[3] Raemy A, Meylan M, Casati S, Gaia V, Berchtold B, Boss R, Wyder A, Graber HU. (2013). Phenotypic and genotypic identification of streptococci and related bacteria isolated from bovine intramammary infections. Acta Vet Scand. 2013 Jul 18;55(1):53. doi: 10.1186/1751-0147-55-53. PMID: 23866930; PMCID: PMC3723560.

[4] Wang, G., Clark, C.G. and Rodgers, F.G., 2002. Detection in Escherichia coli of the genes encoding the major virulence factors, the genes defining the O157:H7 serotype, and components of the type 2 Shiga toxin family by multiplex PCR. Journal of Clinical Microbiology, 40, 3613–3619.

[5] Brisse S, and Verhoef J. (2001). Phylogenetic diversity of Klebsiella pneumonia and Klebsiella oxytoca clinical isolates revealed by randomly amplified polymorphic DNA, gyrA and parC genes sequencing and automated ribotyping. Int J Syst Evolut Microbiol 2001; 51(3):915–24; https://doi.org/10.1099/00207713-51-3-915.
