# Supplementary material for: The association between the use of dry cow therapy and bacteriological cure after calving and the development of phenotypic antimicrobial resistance on Egyptian dairy farms
Source: PLoS One. 2026 Apr 1;21(4):e0345646. doi: 10.1371/journal.pone.0345646 (PMC13043046; doi:10.1371/journal.pone.0345646)
Supplement: S2 Table — (DOCX) [file pone.0345646.s002.docx]

**Table S2.** The percentage of bacterial no growth, pure colonies, mixed infections, and contaminated milk samples collected at dry off and after freshening.

| Culture results | Dry off milk samples | | | Fresh milk samples | | |
| --- | --- | --- | --- | --- | --- | --- |
|  | Fall/Winter (%) | Spring/Summer (%) | Total (%) | Fall/Winter (%) | Spring/Summer (%) | Total (%) |
| No Growth | 31 | 16 | 23 | 20 | 26 | 23 |
| Pure colony | 63 | 76 | 70 | 71 | 65 | 68 |
| Mixed infections | 5 | 7 | 6 | 9 | 7 | 8 |
| Contaminated | 1 | 1 | 1 | 0 | 2 | 1 |
