# Supplementary material for: The association between the use of dry cow therapy and bacteriological cure after calving and the development of phenotypic antimicrobial resistance on Egyptian dairy farms
Source: PLoS One. 2026 Apr 1;21(4):e0345646. doi: 10.1371/journal.pone.0345646 (PMC13043046; doi:10.1371/journal.pone.0345646)
Supplement: S3 Table — (DOCX) [file pone.0345646.s003.docx]

**Table S3.** The percentage of different bacterial isolates isolated from clinical mastitis milk samples during the first 60 days in milk during the Fall/Winter and Spring/Summer seasons.

| Bacterial Isolates | Mastitis milk samples | | |
| --- | --- | --- | --- |
|  | Fall/Winter  (%) | Spring/Summer  (%) | Total (%) |
| *Staphylococcus species* | 29.1 | 29.0 | 29.0 |
| *Staphylococcus aureus* | 41.9 | 32.3 | 37.1 |
| *E. coli* | 29.0 | 29.0 | 29.0 |
| *Klebsiella species* | 0 | 0 | 0 |
| *Streptococcus agalactiae* | 0 | 9.7 | 4.9 |
| *Streptococcus dysgalactiae* | 0 | 0 | 0 |
