# Supplementary material for: The association between the use of dry cow therapy and bacteriological cure after calving and the development of phenotypic antimicrobial resistance on Egyptian dairy farms
Source: PLoS One. 2026 Apr 1;21(4):e0345646. doi: 10.1371/journal.pone.0345646 (PMC13043046; doi:10.1371/journal.pone.0345646)
Supplement: S4 Table — (DOCX) [file pone.0345646.s004.docx]

**Table S4.** The percentage of different bacterial isolates isolated from clinical mastitis milk samples during the first 60 days for different treatment groups

| Bacterial Isolates | Mastitis milk samples | | | | |
| --- | --- | --- | --- | --- | --- |
|  | Control* | AB** | ITS*** | AB&ITS**** | Total |
| *Staphylococcus species* | 8.06 | 4.84 | 8.06 | 8.06 | 29.03 |
| *Staphylococcus aureus* | 9.68 | 8.06 | 11.29 | 8.06 | 37.10 |
| *E. coli* | 8.06 | 1.61 | 6.45 | 12.90 | 29.03 |
| *Streptococcus agalactiae* | 0.00 | 0.00 | 3.23 | 1.61 | 4.84 |

* Control: cows received no treatment

**Cows received intramammary antibiotic tubes only

***Cows received internal teat sealants only.

****Cows received intramammary antibiotic tubes and internal teat sealants
