# Supplementary material for: The association between the use of dry cow therapy and bacteriological cure after calving and the development of phenotypic antimicrobial resistance on Egyptian dairy farms
Source: PLoS One. 2026 Apr 1;21(4):e0345646. doi: 10.1371/journal.pone.0345646 (PMC13043046; doi:10.1371/journal.pone.0345646)
Supplement: S5 Table — (DOCX) [file pone.0345646.s005.docx]

**Table S5.** The percentage of mixed infections, by bacterial species, as isolated from milk samples at collected at dry off and after calving from two Egyptian dairies over the Fall/Winter and Spring/Summer seasons.

| Bacterial Isolates | Milk samples | | | |
| --- | --- | --- | --- | --- |
|  | Fall/Winter | | Spring/Summer | |
|  | Isolate 1 (%) | Isolate 2 (%) | Isolate 1 | Isolate 2 |
| *Staphylococcus species* | 31 | 0 | 56 | 15 |
| *Staphylococcus aureus* | 69 | 0 | 26 | 0 |
| *E. coli* | 0 | 46 | 0 | 52 |
| *Klebsiella species* | 0 | 27 | 0 | 22 |
| *Streptococcus agalactiae* | 0 | 19 | 18 | 4 |
| *Streptococcus dysgalactiae* | 0 | 4 | 0 | 7 |
| *Streptococcus uberis* | 0 | 4 | 0 | 0 |
