# Supplementary material for: The association between the use of dry cow therapy and bacteriological cure after calving and the development of phenotypic antimicrobial resistance on Egyptian dairy farms
Source: PLoS One. 2026 Apr 1;21(4):e0345646. doi: 10.1371/journal.pone.0345646 (PMC13043046; doi:10.1371/journal.pone.0345646)
Supplement: S6 Table — (DOCX) [file pone.0345646.s006.docx]

Table S6. The percentage of isolates at each minimum inhibitory concentration (MIC) for different antimicrobials for the *Staphylococcus aureus* isolates from the dry off milk samples for the group that received intramammary antibiotics.

| Antimicrobial/MIC values (µg/mL) | 0.12 | 0.25 | 0.5 | 1 | 2 | 4 | 8 | 16 | 32 | 64 | 128 | 256 | MC 50 | MC 90 |
| --- | --- | --- | --- | --- | --- | --- | --- | --- | --- | --- | --- | --- | --- | --- |
| Ampicillin | 39 | 35 | **9** | 0 | 13 | 0 | 4 |  |  |  |  |  | 0.25 | 2.00 |
| Penicillin | 48 | **13** | 13 | 4 | 4 | 0 | 17 |  |  |  |  |  | 0.25 | 8.00 |
| Erythromycin |  | 52 | 22 | 4 | 0 | **22** |  |  |  |  |  |  | 0.25 | 4.00 |
| Ceftiofur |  |  | 39 | 39 | 4 | **17** |  |  |  |  |  |  | 1.00 | 4.00 |
| Pirlamycin |  |  | 74 | 4 | 4 | **17** |  |  |  |  |  |  | 0.50 | 4.00 |
| Pencillin/Novobiocin |  |  |  | 96 | 4 | **0** | 0 |  |  |  |  |  | 1.00 | 1.00 |
| Tetracycline |  |  |  | 70 | 4 | 9 | **17** |  |  |  |  |  | 1.00 | 8.00 |
| Cephalothin |  |  |  |  | 91 | 0 | 4 | 4 |  |  |  |  | 2.00 | 2.00 |
| Oxacillin |  |  |  |  | 87 | **13** |  |  |  |  |  |  | 2.00 | 4.00 |
| Sulfadimethoxine |  |  |  |  |  |  |  |  | 43 | 13 | 4 | **39** | 64.00 | ≥ 256 |
