# Supplementary material for: The association between the use of dry cow therapy and bacteriological cure after calving and the development of phenotypic antimicrobial resistance on Egyptian dairy farms
Source: PLoS One. 2026 Apr 1;21(4):e0345646. doi: 10.1371/journal.pone.0345646 (PMC13043046; doi:10.1371/journal.pone.0345646)
Supplement: S8 Table — (DOCX) [file pone.0345646.s008.docx]

Table S8. The percentage of isolates at each minimum inhibitory concentration (MIC) for different antimicrobials for the *Staphylococcus aureus* isolates from the dry off milk samples for the group that received intramammary antibiotics and internal teat sealants at dry off.

| Antimicrobial/MIC values (µg/mL) | 0.12 | 0.25 | 0.5 | 1 | 2 | 4 | 8 | 16 | 32 | 64 | 128 | 256 | MC 50 | MC 90 |
| --- | --- | --- | --- | --- | --- | --- | --- | --- | --- | --- | --- | --- | --- | --- |
| Ampicillin | 49 | 34 | **0** | 3 | 9 | 3 | 3 |  |  |  |  |  | 0.25 | 2.00 |
| Penicillin | 51 | **23** | 3 | 6 | 0 | 3 | 14 |  |  |  |  |  | 0.12 | 8.00 |
| Erytheromycin |  | 37 | 37 | 0 | 0 | **26** |  |  |  |  |  |  | 0.50 | 4.00 |
| Ceftiofur |  |  | 51 | 31 | 3 | **14** |  |  |  |  |  |  | 0.50 | 4.00 |
| Pirlamycin |  |  | 60 | 14 | 3 | **23** |  |  |  |  |  |  | 0.50 | 4.00 |
| Pencillin/Novobiocin |  |  |  | 86 | 11 | **0** | 3 |  |  |  |  |  | 1.00 | 2.00 |
| Tetracycline |  |  |  | 86 | 6 | 0 | **9** |  |  |  |  |  | 1.00 | 2.00 |
| Cepahlothin |  |  |  |  | 86 | 3 | 0 | 11 |  |  |  |  | 2.00 | 16.00 |
| Oxacillin |  |  |  |  | 71 | **29** |  |  |  |  |  |  | 2.00 | 4.00 |
| Sulfadimethoxine |  |  |  |  |  |  |  |  | 43 | 17 | 3 | **37** | 64.00 | ≥ 256 |
