# Supplementary material for: The association between the use of dry cow therapy and bacteriological cure after calving and the development of phenotypic antimicrobial resistance on Egyptian dairy farms
Source: PLoS One. 2026 Apr 1;21(4):e0345646. doi: 10.1371/journal.pone.0345646 (PMC13043046; doi:10.1371/journal.pone.0345646)
Supplement: S10 Table — (DOCX) [file pone.0345646.s010.docx]

Table S10. The percentage of isolates at each minimum inhibitory concentration (MIC) for different antimicrobials for the *Staphylococcus aureus* isolates from the dry off milk samples for the group that received internal teat sealants at dry off.

| Antimicrobial/MIC values (µg/mL) | 0.12 | 0.25 | 0.5 | 1 | 2 | 4 | 8 | 16 | 32 | 64 | 128 | 256 | MC 50 | MC 90 |
| --- | --- | --- | --- | --- | --- | --- | --- | --- | --- | --- | --- | --- | --- | --- |
| Ampicillin | 38 | 38 | **9** | 6 | 0 | 0 | 9 |  |  |  |  |  | 0.25 | 1.00 |
| Penicillin | 47 | **26** | 6 | 9 | 0 | 3 | 9 |  |  |  |  |  | 0.25 | 1.00 |
| Erytheromycin |  | 44 | 26 | 9 | 3 | **18** |  |  |  |  |  |  | 0.50 | 4.00 |
| Ceftiofur |  |  | 26 | 47 | 9 | **18** |  |  |  |  |  |  | 1.00 | 4.00 |
| Pirlamycin |  |  | 76 | 9 | 0 | **15** |  |  |  |  |  |  | 1.00 | 4.00 |
| Pencillin/Novobiocin |  |  |  | 94 | 6 | **0** | 0 |  |  |  |  |  | 1.00 | 1.00 |
| Tetracycline |  |  |  | 85 | 0 | 0 | **15** |  |  |  |  |  | 1.00 | 8.00 |
| Cepahlothin |  |  |  |  | 82 | 12 | 3 | 3 |  |  |  |  | 2.00 | 4.00 |
| Oxacillin |  |  |  |  | 88 | **12** |  |  |  |  |  |  | 2.00 | 4.00 |
| Sulfadimethoxine |  |  |  |  |  |  |  |  | 44 | 6 | 0 | **50** | 64.00 | ≥ 256 |
