# Supplementary material for: The association between the use of dry cow therapy and bacteriological cure after calving and the development of phenotypic antimicrobial resistance on Egyptian dairy farms
Source: PLoS One. 2026 Apr 1;21(4):e0345646. doi: 10.1371/journal.pone.0345646 (PMC13043046; doi:10.1371/journal.pone.0345646)
Supplement: S12 Table — (DOCX) [file pone.0345646.s012.docx]

Table S12. The percentage of isolates at each minimum inhibitory concentration (MIC) for different antimicrobials for the *Staphylococcus aureus* isolates from the dry off milk samples collected during the Fall/Winter season for the control group.

| Antimicrobial/MIC values (µg/mL) | 0.12 | 0.25 | 0.5 | 1 | 2 | 4 | 8 | 16 | 32 | 64 | 128 | 256 | MC 50 | MC 90 |
| --- | --- | --- | --- | --- | --- | --- | --- | --- | --- | --- | --- | --- | --- | --- |
| Ampicillin | 31 | 35 | **0** | 19 | 4 | 8 | 4 |  |  |  |  |  | 0.25 | 4.00 |
| Penicillin | 46 | **19** | 15 | 4 | 8 | 0 | 8 |  |  |  |  |  | 0.25 | 2.00 |
| Erythromycin |  | 42 | 35 | 0 | 0 | **23** |  |  |  |  |  |  | 0.50 | 4.00 |
| Ceftiofur |  |  | 31 | 58 | 8 | **4** |  |  |  |  |  |  | 1.00 | 2.00 |
| Pirlamycin |  |  | 69 | 4 | 8 | **19** |  |  |  |  |  |  | 0.50 | 4.00 |
| Pencillin/Novobiocin |  |  |  | 96 | 4 | **0** | 0 |  |  |  |  |  | 1.00 | 1.00 |
| Tetracycline |  |  |  | 77 | 8 | 0 | **15** |  |  |  |  |  | 1.00 | 8.00 |
| Cephalothin |  |  |  |  | 96 | 4 | 0 | 0 |  |  |  |  | 2.00 | 2.00 |
| Oxacillin |  |  |  |  | 88 | **12** |  |  |  |  |  |  | 2.00 | 4.00 |
| Sulfadimethoxine |  |  |  |  |  |  |  |  | 46 | 12 | 0 | **42** | 64.00 | ≥ 256 |
