# Supplementary material for: The association between the use of dry cow therapy and bacteriological cure after calving and the development of phenotypic antimicrobial resistance on Egyptian dairy farms
Source: PLoS One. 2026 Apr 1;21(4):e0345646. doi: 10.1371/journal.pone.0345646 (PMC13043046; doi:10.1371/journal.pone.0345646)
Supplement: S13 Table — (DOCX) [file pone.0345646.s013.docx]

Table S13. The percentage of isolates at each minimum inhibitory concentration (MIC) for different antimicrobials for the *Staphylococcus aureus* isolates from the fresh milk samples collected during the Fall/Winter season for the control group.

| Antimicrobial/MIC values (µg/mL) | 0.12 | 0.25 | 0.5 | 1 | 2 | 4 | 8 | 16 | 32 | 64 | 128 | 256 | MC 50 | MC 90 |
| --- | --- | --- | --- | --- | --- | --- | --- | --- | --- | --- | --- | --- | --- | --- |
| Ampicillin | 21 | 10 | **14** | 10 | 7 | 7 | 31 |  |  |  |  |  | 1.00 | 8.00 |
| Penicillin | 14 | **17** | 10 | 3 | 7 | 3 | 45 |  |  |  |  |  | 2.00 | 8.00 |
| Erythromycin |  | 41 | 31 | 3 | 0 | **24** |  |  |  |  |  |  | 0.50 | 4.00 |
| Ceftiofur |  |  | 10 | 34 | 17 | **38** |  |  |  |  |  |  | 2.00 | 4.00 |
| Pirlamycin |  |  | 83 | 0 | 3 | **14** |  |  |  |  |  |  | 0.50 | 4.00 |
| Pencillin/Novobiocin |  |  |  | 90 | 7 | **0** | 3 |  |  |  |  |  | 1.00 | 1.00 |
| Tetracycline |  |  |  | 48 | 3 | 0 | **48** |  |  |  |  |  | 2.00 | 8.00 |
| Cephalothin |  |  |  |  | 72 | 0 | 0 | 28 |  |  |  |  | 2.00 | 16.00 |
| Oxacillin |  |  |  |  | 55 | **45** |  |  |  |  |  |  | 2.00 | 4.00 |
| Sulfadimethoxine |  |  |  |  |  |  |  |  | 28 | 10 | 7 | **55** | ≥ 256 | ≥ 256 |
